# Quality of life outcomes for informal carers of long-term care service users in Austria, England and Finland

**DOI:** 10.1007/s11136-024-03711-2

**Published:** 2024-06-22

**Authors:** Ismo Linnosmaa, Lien Nguyen, Hanna Jokimäki, Eirini-Christina Saloniki, Juliette Malley, Birgit Trukeschitz, Assma Hajji, Julien Forder

**Affiliations:** 1 Finnish Institute for Health and Welfare (THL), Helsinki, Finland; 2 Department of Health and Social Management, University of Eastern Finland, Kuopio, Finland; 3 Statistics Finland, Helsinki, Finland; 4 Department of Primary Care and Population Health, University College London, London, UK; 5 NIHR Applied Research Collaboration North Thames, London, UK; 6 London School of Economics and Political Science, Care Policy and Evaluation Centre, London, UK; 7 WU Vienna University of Economics and Business, Research Institute for Economics of Aging, Vienna, Austria; 8 Personal Social Services Research Unit, University of Kent, Canterbury, Kent, UK

**Keywords:** Informal care, Quality of life, Risk-adjustment, Comparative analysis, Long-term care

## Abstract

**Purpose:**

The provision and funding of long-term care (LTC) for older people varies between European countries. Despite differences, there is limited information about the comparative performance of LTC systems in Europe. In this study, we compared quality of life (QoL) of informal carers of home care service users in Austria, England and Finland.

**Methods:**

Informal carers were surveyed in Austria, England and Finland. The study data (n = 835) contained information on social care-related quality of life (SCRQoL) associated with the ASCOT-Carer measure, and characteristics of carers and care recipients from each country. We applied risk-adjustment methods using a fractional regression model to produce risk-adjusted SCRQoL scores for the comparative analysis. In a sensitivity analysis, we applied multiple imputation to missing data to validate our findings.

**Results:**

We found that the mean values of the risk-adjusted SCRQoL of informal carers in England were 1.4–2.9% and 0.3–0.5% higher than in Finland and Austria, and the mean values of the risk-adjusted SCRQoL of carers in Austria were 0.8–2.7% higher than in Finland. Differences in the mean values of the country-specific risk-adjusted SCRQoL scores were small and statistically non-significant. English informal carers were less healthy and co-resided with care resipients more often than carers in Austria or Finland.

**Conclusion:**

Small differences between the risk-adjusted SCRQoL scores between Austria, England and Finland are consistent with the observation that the countries provide different types of support for informal carers. Our results help local and national decision-makers in these countries to benchmark their informal care support systems.

**Supplementary Information:**

The online version contains supplementary material available at 10.1007/s11136-024-03711-2.

## Introduction

European countries differ in how they organise the funding and provision of long-term care (LTC) for older people. The extent to which LTC provision relies on formal or informal care varies between European countries [1, 2]. Disparities also exist in the share of GDP spent on LTC services; in the share of total LTC expenditure covered by public expenditure; and in what degree LTC benefits are provided in cash or in-kind [3, 4].

When differences in the provision and funding of LTC services are systematically linked to relevant service user outcomes, policy makers and researchers can use them to learn more about the performance of LTC systems in Europe. Empirical evidence on the comparative performance of LTC systems can result in best practice models that national and local policy makers can utilise to improve the effectiveness and cost-effectiveness of services and support systems. While such information on the performance of healthcare systems in European countries is increasingly available [5–8], similar information on the performance of LTC services and support systems for informal carers is very limited [9, 10].

Informal care—unpaid care provided to care recipients by their spouses, children, relatives, neighbours or friends [11]—plays a significant role in LTC provision. Fujisawa and Colombo [2] highlighted that in Italy, the Netherlands, England and Northern Ireland, Spain and the USA, the number of informal carers (hereafter also carers or caregivers) far exceeds the number of care workers providing formal services. Informal care is socially beneficial due to its potential to substitute for formal services, such as institutional or home care services [12, 13]. However, there are opportunity costs and health consequences associated with informal care provision. Providing informal care reduces earnings and the likelihood of employment of female carers [14] and increases the likelihood of mental health problems [15]. To alleviate such problems, several countries have introduced cash benefits and services to reduce the care burden and to support carers’ health and quality of life (QoL) [3, 16].

We compared the QoL of informal carers caring for older people receiving home care in Austria, England and Finland. Each country has developed a system of informal carer support. However, the organisation, funding and culture of the LTC system in these countries are different, with a universal entitlement system in Finland, a universal cash benefit and income-related public co-payments for care services in Austria, and a means-tested system in England. We analysed QoL variation between informal carers that was related to cross-country differences in support for informal carers as opposed to cross- and within-country QoL variation relating to carer and care recipient characteristics, the latter of which are hereafter called risk-adjustment factors. Even after risk-adjustment [17], we expected informal carers’ QoL to differ between these countries, and this is the hypothesis we explored in this study.

To operationalize QoL, we measured social care-related quality of life (SCRQoL) of informal carers using Adult Social Care Outcomes Toolkit (ASCOT) for carers (hereafter ASCOT-Carer) [18, 19]. The current study built on Identifying the Impact of Adult Social Care (IIASC) study that sought to explore the determinants of SCRQoL using ASCOT [20]. To compare carers’ QoL between countries with different support systems, we collected data on the SCRQoL of informal carers in Austria and Finland using ASCOT-Carer and supplemented this with the anonymised English IIASC data. Our cross-section data set is rich in descriptive characteristics of carers and care recipients, allowing us to control for risk-adjustment factors when comparing carers’ SCRQoL in the three countries. Thus, we investigated (i) what risk-adjustment factors explain the variation of SCRQoL of informal carers in Austria, England and Finland; (ii) whether risk-adjusted SCRQoLs of informal carers differ between Austria, England and Finland.

## Support for informal carers in Austria, England and Finland

Carers’ SCRQoL can be influenced both by services and benefits provided to themselves and care recipients and the characteristics of carers and care recipients [21–24]. Below, we describe the principles and extent of support systems for informal carers in Austria, England and Finland that are expected to explain SCRQoL differences between the countries. As our data were collected between 2012 and 2017 (Sect. Data), we refer to the time of introduction of changes in country-specific informal care policies whenever possible. Trukeschitz et al. [9] describe more generally the LTC systems in these three countries.

The Austrian LTC system builds on supported family care, which is extended with the support of the purchasing power of the care recipients and a service infrastructure for home care and temporary and permanent institutional care [25, 26]. Services and benefits available for informal carers are family hospice leave, care leave and part-time work options, respite care, improved sickness insurance coverage, psychosocial support and counselling [26] (Table 1). While this range of benefits and services for informal carers seems comprehensive, eligibility rules are tight [26] and take up is very low [27]. For most benefits, access is restricted to family carers caring for LTC allowance beneficiaries who need at least 120 h of care per month. At the time of data collection, no federal carer allowance was available in Austria. Since July 2023, informal carers −caring for a partner or close relative who receives LTC allowance at level 4 or higher− can apply for an income-tested bonus payment if they provide most of the care work [28]. In addition, there are regional initiatives to formalise informal care by introducing employment schemes for informal carers [26].

In England, informal carers are eligible for a carer allowance if they care for someone at least 35 h per week. The carer allowance is means-tested and available to informal carers with income below the defined weekly income limit. Eligibility is also dependent on the disability status of the care recipient, who must be in receipt of a disability benefit [29]. Services and benefits for informal carers include needs assessments (right since 2014) and care plans, care leave, respite care, advice on accessing health services, and flexible working arrangements (Table 1) [30]. Carers’ eligibility for services and benefits is defined in the care and support regulations [31]. Some local authorities also organise well-being support (such as peer support) for carers, and the voluntary sector provides support services that may incur out-of-pocket payments for carers.

In Finland, support for informal care is considered a formal service consistent with the Nordic model of public services [32]. Support for informal care can be granted if the care recipient’s need for home-based care, the informal carer’s capability to carry out care duties, and the suitability of the care environment for care provision have been confirmed. Services and benefits available for informal carers include need assessments and care plans, carer allowance, care leave, respite care for the duration of care leave, medical examinations and carer training (since 2016) as well as other services that support informal carers and enhance the well-being of care recipients [33] (Table 1).

**Table 1:** Informal carer support in Austria, England and Finland

	Carer allowance	Available services and benefits	Eligibility and access
**Austria**	No carer allowance	Family hospice leave Care leave and part-time employment options Respite care Improved sickness insurance coverage Psychosocial support and counselling Regional initiative (in Burgenland) to develop employment schemes for informal carers since 2019	Access to most of the benefits is restricted to family carers caring for LTC allowance beneficiaries who need at least 120 h of care per month (level 3).
**England**	Yes	Need assessments Care plans Care leave Respite care Advice on accessing health services Flexible working hours	Carers are eligible for a means-tested carer allowance if they spend at least 35 h a week caring for someone, and for services and benefits if they meet eligibility criteria defined in the care and support regulations [30].
**Finland**	Yes	Need assessments Care plans Care leave Respite care Medical examinations Training in care tasks Other services supporting informal care and well-being of care recipients	Support for informal care can be granted if care recipients’ need for home-based care, informal carer’s capability to accept care duties and the suitability of the care environment are confirmed.

Sources: Trukeschitz, Österle and Schneider [26], Anderson et al. [29], Foley et al. [30], Laki omaishoidon tuesta [33]

## Data and methods

### Data

We used personal standardised survey interview data from three countries. English data were collected in the IIASC project from 2012 to 2013 [20] and Austrian and Finnish data in EXCELC (Exploring Comparative Effectiveness and Efficiency in Long-term Care, www.excelc.eu) project from April 2016–October 2017. To ensure comparability of the collected country-specific data, questionnaires and survey methods used in the EXCELC project were based on those used in the IIASC project [20]. This approach allowed us to merge the EXCELC data with the (anonymised) IIASC data and to maintain consistent specifications of the variables in three country-specific datasets. Given different times of data collection, we compare the performance of informal carers’ support system in England in 2012–2013 and in Austria and Finland in 2016–2017.

Target populations in both above-mentioned projects were LTC service users in non-institutional care aged at least 55 years and their informal carers. Service users were invited to participate in the study by sending them invitation letters. Carers were recruited mainly via service users’ interviews [20, 34, 35]. Some carers were also recruited via administrative records of municipalities or health and social care organisations and regions or during care workers’ regular home care visits [34]. When service users had several carers, we interviewed the person providing the largest share of assistance. Written informed consents were obtained from both participating service users and carers.

For the comparative analysis, a three-country data set with selected relevant variables was generated. Carers’ SCRQoL was measured using the ASCOT-Carer four-level interview schedule (INT4) [18], which has been translated into German [35] and Finnish [34]. The ASCOT-Carer instrument has seven domains: ‘occupation’, ‘control over daily life’, ‘looking after yourself’, ‘personal safety’, ‘social participation and involvement’, ‘space and time to be yourself’, and ‘feeling supported and encouraged’. Each domain has four levels corresponding to the intensity of need for support and care: ‘ideal state’ (level 3, top level), ‘no needs’ (level 2), ‘some needs’ (level 1) and ‘high needs’ (level 0, bottom level). Carers’ SCRQoL was measured based on the current receipt of support and services for carers and service users (current SCRQoL) and on the hypothetical situation of not having received the current support and services (expected SCRQoL) [19]. Because preference-weighted SCRQoL scores reflect country-specific valuations of the ASCOT-Carer domain levels, they were applied. SCRQoL scores were computed using country-specific preference weights and were scaled so that they vary from 0 to 1, where zero (one) refers to the lowest (highest) possible SCRQoL [36–38].

The analysis data set contained information about country variables, carers’ socio-demographic background (such as age, sex, marital status, education, occupation, household financial situation), carers’ health (self-assessed health (SAH), EQ-5D-3 L, long-term illnesses), care burden (number of care tasks and hours spent on care tasks, duration of care), care environment (co-residence with care recipient, suitability of home for care) and carers’ social contacts (frequency of social contacts). Data also contained basic information about care recipients (such as age, sex and cognitive health).

### Risk adjustment

To compare carers’ SCRQoL in three countries, we applied risk-adjustment methods [17, 39–42]. A fractional regression model was used to model the relationship between current SCRQoL and risk-adjustment factors [43]. The model is suitable to analyse dependent variables that are bounded between zero and one and it has been applied to analyse ASCOT data [44]. An alternative model to consider would be the beta regression model [45], which is, however, restrictive as it would exclude the endpoints that were present in the current SCRQoL variable.

The estimated fractional regression model can be written as:

1 $$E\left({y}_{ij}|{x}_{ij}\right)=G\left({x}_{ij}^{{\prime }}{\beta }\right),$$

where the dependent variable *y*_*ij*_ measures the current SCRQoL of carer *i* in country *j*, the vector *x*_*ij*_ contains risk-adjustment factors for carer *i* in country *j*, and parameters β are regression coefficients (including the constant term).

The parameters of Model (1) and their heteroscedastic-robust standard errors were estimated using quasi-maximum likelihood techniques [43]. We used a probit specification of Model (1) because it provided a better fit to our data than a logit specification in all estimated risk-adjustment models. To measure the proportion of variation in informal carers’ current SCRQoL that was explained by risk-adjustment factors, we used Efron’s R^2^ and pseudo R^2^, where the former is an analogous measure to the coefficient of determination (R^2^) in the linear regression model [46, 47]. When choosing suitable specifications for the risk-adjustment model for the comparative analysis, we used the Wald-test for linear restrictions [48].

The indirect standardisation method [39] was applied to compute the risk-adjusted current SCRQoL in each country. We computed O/E-ratios, where the numerator O refers to the observed current SCRQoL and the denominator E to the expected current SCRQoL of carers, as predicted by Model (1). O/E-ratios were multiplied by the sample average of the current SCRQoL to obtain the risk-adjusted current SCRQoLs.

### Risk-adjustment models and factors

We estimated three specifications of Model (1) using informal carer- and care recipient-related risk-adjustment factors as explanatory variables:

- Model A: Expected SCRQoL, carers’ demographic characteristics and health.
- Model B: Covariates included in Model A + carers’ co-residence.
- Model C: Covariates included in Model B + care recipients’ characteristics.

The expected SCRQoL was included in all risk-adjustment models as an explanatory variable. The inclusion was justified because the expected SCRQoL may contain relevant information about unobserved factors, such as unobserved need factors [49]. Variables affecting carers’ eligibility for informal care support or the amount of support received were excluded from the risk-adjustment models because they may capture the effects of country-specific support systems. Such variables were care burden, carers’ mental health, income and education, and the care recipients’ cognitive health.

Model A also included the carers’ sex (= 1 if the carer was female), age and health as control variables to explore the variation of the current SCRQoL [44, 49, 50]. To allow for a non-linear relationship between carers’ age and current SCRQoL, age was measured using a three-level categorical variable: 0 = Younger than 65 years, 1 = 65−74 years old, and 2 = 75 years old or older. The original five-level SAH variable was recoded as: 0 = Very good or good health, 1 = Fair health, and 2 = Very bad or bad health.

Model B included further co-residence (= 1 if the carer lived with the care recipient and 0 otherwise) as a variable capturing carers’ care burden and captivity [18]. Finally, we added care recipients’ age and sex (= 1 if the care recipient was female) to Model C to allow for carer-related risk-adjustment factors. Care recipients’ age was coded as: 0 = Younger than 74 years and 1 = 75 years old or older (Table 2).

**Table 2:** Descriptive results

	Full sample (*n* = 829)		England (*n* = 237)		Finland (*n* = 249)		Austria (*n* = 343)		
**Panel A**: Categorical variables	obs.	%	obs.	%	obs.	%	obs.	%	*p*-value^a^
**I Informal carer**:									
**Sex**									< 0.001
Male	288	34.74	111	46.84	66	26.51	111	32.36	
Female	541	65.26	126	53.16	183	73.49	232	67.64	
**Age**									0.773
Younger than 64 years	378	45.60	108	45.57	106	42.57	164	47.81	
65–74 years	238	28.71	70	29.54	74	29.72	94	27.41	
75 years old or older	212	25.57	59	24.89	69	27.71	84	24.49	
Missing	1	0.12	0	0.00	0	0.00	1	0.29	
**Self-assessed health**									< 0.001
Very good or good	453	54.64	103	43.46	145	58.23	205	59.77	
Fair	286	34.50	92	38.82	79	31.73	115	33.53	
Very bad or bad	90	10.86	42	17.72	25	10.04	23	6.71	
**Co-residence**									< 0.001
No	323	38.96	52	21.94	146	58.63	125	36.44	
Yes	505	60.92	185	78.06	103	41.37	217	63.27	
Missing	1	0.12	0	0.00	0	0.00	1	0.29	
**II Care recipient**:									
**Sex**									< 0.001
Male	294	35.46	94	39.66	75	30.12	125	36.44	
Female	497	59.95	143	60.34	136	54.62	218	63.56	
Missing	38	4.58	0	0.00	38	15.26	0	0.00	
**Age**									< 0.001
Younger than 74 years	214	25.82	125	52.74	28	11.24	61	17.78	
75 years old or older	577	69.60	111	46.84	184	73.90	282	82.22	
Missing	38	4.58	1	0.42	37	14.48	0	0.00	
**Panel B**: Continuous variables									
**Current SCRQoL**	**Full Sample** (***n*** = **805**)		**England** (***n*** = **234**)		**Finland** (***n*** = **243**)		**Austria** (***n*** = **328**)		
	Mean	95% CI^c^	Mean	95% CI^c^	Mean	95% CI^c^	Mean	95% CI^c^	*p*-value^b^
	0.730	0.714; 0.745	0.734	0.705; 0.763	0.719	0.691; 0.748	0.735	0.710; 0.759	England vs. Finland: 0.485; England vs. Austria: 0.973; Finland vs. Austria: 0.434
**Expected SCRQoL**	**Full sample** (***n*** = **781**)		**England** (***n*** = **233**)		**Finland** (***n*** = **234**)		**Austria** (***n*** = **314**)		
	Mean	95% CI^c^	Mean	95% CI^c^	Mean	95% CI^c^	Mean	95% CI^c^	*p*-value^b^
	0.428	0.408; 0.448	0.497	0.458; 0.535	0.397	0.362; 0.431	0.401	0.370; 0.430	England vs. Finland: <0.001; England vs. Austria: <0.001; Finland vs. Austria: 0.864

^a^*p*-value of the Pearson’s χ2-test for independence

^b^*p*-value of the two-sided t-test for the equality of means

^c^ 95% confidence interval: Lower; Upper

### Imputation of missing values

In the sensitivity analysis, missing values were imputed by multiple imputation with chained equations [51]. Our data contained 90 observations with missing values in at least one of the analysis variables. Current SCRQoL, country dummies, risk-adjustment factors and the number of care tasks were included as predictors in the imputation models. We created 50 complete data sets and re-estimated Model (1) using Rubin’s estimation techniques [52]. To evaluate the risk-adjusted SCRQoL of carers using the complete data sets, we computed the predicted current SCRQoL as the average of predictions of Model (1) from the imputed data sets [53] and used the current SCRQoL observations in our data (*n* = 805, Table 2) to compute the O/E ratio and the carers’ current risk-adjusted SCRQoL.

## Results

### Descriptive findings

Our data set had 344 observations from Austria, 237 observations from England, and 254 observations from Finland (*n* = 835). We excluded six observations due to missing information about carers’ characteristics. In the remaining sample (*n* = 829), 90 observations had missing values for at least one of the analysis variables, reducing the sample size to 739 observations (308 from Austria, 231 from England, and 200 from Finland).

The mean value of informal carers’ current SCRQoL was lower in Finland (0.719, *n* = 243) than in England (0.734, *n* = 234) or Austria (0.735, *n* = 328). However, pairwise comparisons showed that differences in the mean values were non-significant. (Table 2) Descriptive findings also indicated several differences in the risk-adjustment factors between three countries.

The biggest cross-country differences in the risk-adjustment factors were observed regarding informal carers’ sex, SAH, co-residence, care recipients’ age, and expected SCRQoL (Table 2). In Finland, about 73% of carers were female, while the corresponding figures in England and Austria were 53% and 68%. Finnish and Austrian informal carers assessed their health to be better than English carers did. The fraction of English carers self-reporting either good or very good health was 43%, while in Finland and Austria this proportion of carers varied from 58 to 60%.

Co-residence of informal carers with care recipients was more common in England than in Austria or Finland. Regarding care recipient’s age, more than 73% of carers in Finland and Austria but only about 47% of carers in England cared for a person who was older than 74 years. Finally, the mean value of the carers’ expected SCRQoL in England clearly exceeded the corresponding figures in Finland or Austria (Table 2).

### Results of the risk-adjustment models using data without missing values

According to Efron’s R^2^, the risk-adjustment factors in Models A−C explain 43−44% of the variation in carers’ current SCRQoL. The Wald-test rejected Model A in favour of Models B and C, but it did not reject Model B in favour of Model C (Table 3). Therefore, we used Model B to compute the risk-adjusted current SCRQoLs.

Informal carers’ current SCRQoL was affected by the expected SCRQoL, SAH and co-residence (Table 3). A strong positive association was found between expected SCRQoL and current SCRQoL (*p* < 0.001). The estimated marginal effect indicated that a one unit increase in expected SCRQoL (from the lowest [or poorest] value to the highest [or best] value of SCRQoL) was associated with an increase of 0.409 units in current SCRQoL. The strong positive association is likely to reflect the fact that the expected SCRQoL was related to carers’ unobservable need factors. Poor SAH was associated with a reduction in current SCRQoL. On average, the SCRQoL of carers with bad or very bad (fair) SAH was 0.163 (0.104) units lower than that of carers with good or very good SAH. In addition, carers’ co-residence was negatively associated with current SCRQoL (*p* < 0.001).

On average, compared to the expected current SCRQoL (as predicted by Model B), the observed current SCRQoL of carers in England (Austria) was 0.7% (0.2%) higher, but the corresponding value in Finland was 0.7% lower (Table 3). These results imply that the mean value of risk-adjusted current SCRQoL in England was 0.5% (1.4%) higher than in Austria (Finland), and the mean value of risk-adjusted SCRQoL in Austria was 0.8% higher than the corresponding figure in Finland. However, these differences are small. Pairwise comparisons indicated that none of the differences between the country-specific mean values of the risk-adjusted SCRQoLs was statistically significant (Table 4).

**Table 3:** Results of the risk-adjustment models (*n* = 739)

Variables	Model A (fractional probit)		Model B (fractional probit)		Model C (fractional probit)		Model B (fractional probit)	
	Coefficient	Std. error^a^	Coefficient	Std. error^a^	Coefficient	Std. error^a^	Marginal effect	Std. error^b^
Constant	0.376***	0.062	0.505***	0.067	0.468***	0.095		
**I Informal carer**:								
**Expected SCRQoL**								
Expected SCRQoL	1.430***	0.072	1.402***	0.072	1.406***	0.073	0.409***	0.021
**Sex** (ref: Male)								
Female	-0.034	0.045	-0.070	0.045	-0.070	0.055	-0.020	0.013
**Age** (ref: Younger than 64 years)								
65–74 years old	-0.114*	0.050	-0.060	0.050	-0.059	0.051	-0.017	0.014
75 years old or older	-0.179**	0.053	-0.084	0.056	-0.092	0.062	-0.025	0.016
**Self-assessed health** (ref: Good or very good)								
Fair	-0.376***	0.045	-0.349***	0.046	-0.348***	0.046	-0.104***	0.014
Bad or very bad	-0.575***	0.067	-0.525***	0.068	-0.518***	0.069	-0.163***	0.023
**Co-residence** (ref: No)								
Yes			-0.231***	0.051	-0.220**	0.053	-0.067**	0.014
**II Care recipient**:								
**Sex** (ref: Male)								
Female					0.011	0.053		
**Age** (ref: Younger than 74 years)								
75 years old or older					0.031	0.051		
n	739		739		739			
Log pseudolikelihood	-384.725		-383.007		-382.970			
Pseudo R^2^	0.101		0.105		0.105			
Efron’s R^2^	0.431		0.444		0.444			
**Wald-test of linear restrictions**								
Model A vs. Model B			20.59***					
Model A vs. Model C					21.00***			
Model B vs. Model C					0.48			
**Mean value of the O/E-ratio**:								
England	0.993		1.007		1.010			
Finland	1.011		0.993		0.992			
Austria	1.000		1.002		1.000			

*** *p* < 0.001; **p < 0.01; *p < 0.05

^a^ Robust standard errors

^b^ Standard errors were computed using the delta method

**Table 4:** Mean values of the risk-adjusted current SCRQoLs of carers in England, Austria and Finland (*n* = 739)

Model B was used to compute risk-adjusted SCRQoL				
**Panel I**: Risk-adjusted current SCRQoL	Mean	Std. error		
England	0.735	0.013		
Finland	0.725	0.012		
Austria	0.731	0.012		
**Panel II**: Pairwise testing for differences in risk-adjusted current SCRQoL means	Difference in means	Std. error	t-statistic^a^	*p*-value^b^
England vs. Finland	0.010	0.018	0.563	0.573
England vs. Austria	0.003	0.018	0.1875	0.851
Finland vs. Austria	-0.007	0.017	-0.396	0.692

*** *p* < 0.001; ** *p* < 0.01; * *p* < 0.05

^a^ Appropriate t-statistic was chosen after testing for the equality of variances

^b^*p*-value of a two-sided t-test for the equality of means

### Results of the models using imputed data

Results from Models A−C using the imputed data did not differ from the main findings reported in Table 3 (Table 5). The current SCRQoL of carers had a positive association with the expected SCRQoL and negative associations with fair, bad or very bad SAH and co-residence.

Tables 5 and 6 display the mean values of the O/E-ratios and the risk-adjusted current SCRQoLs. The observed current SCRQoL of carers in England and Austria (Finland) were on average 0.8% and 0.6% higher (2.0% lower) than the expected current SCRQoL as predicted by Model B. The average O/E-ratio in Finland was now lower compared to that from the main analysis because the Finnish mean value of current SCRQoL (Table 2) in the sensitivity analysis (*n* = 805) was smaller than the corresponding Finnish mean value in the main analysis (*n* = 739). As a result, the mean value of the risk-adjusted current SCRQoL of carers in England was 0.3% (2.9%) higher than in Austria (Finland), and the corresponding mean value in Austria exceeded that in Finland by 2.7%. Although the differences in the mean values of the risk-adjusted SCRQoL were somewhat larger after imputation of missing values, pairwise comparisons of the mean values showed that none of the differences differed from zero statistically at *p* < 0.05.

**Table 5:** Estimation results using imputed data sets (*n* = 829)

Variables	Model A (fractional probit)		Model B (fractional probit)		Model C (fractional probit)	
	Coefficient	Std. error^a^	Coefficient	Std. error^a^	Coefficient	Std. error^a^
Constant	0.357***	0.060	0.470***	0.064	0.453***	0.092
**I Informal Carer**:						
**Expected SCRQoL**						
Expected SCRQoL	1.427***	0.070	1.405***	0.070	1.410***	0.070
**Sex** (ref: Male)						
Female	-0.036	0.043	-0.068	0.043	-0.077	0.053
**Age** (ref: Younger than 64 years)						
65–74 years old	-0.084	0.049	-0.036	0.049	-0.037	0.050
75 years old or older	-0.156**	0.050	-0.065	0.053	-0.078	0.059
**Self-assessed health** (ref: Good or very good)						
Fair	-0.375***	0.044	-0.353***	0.044	-0.352***	0.044
Bad or very bad	-0.570***	0.063	-0.524***	0.064	-0.519***	0.065
**Co-residence** (ref: No)						
Yes			-0.212***	0.048	-0.204***	0.050
**II Care recipient**:						
**Sex** (ref: Male)						
Female					-0.006	0.052
**Age** (ref: Younger than 74 years)						
75 years old or older					0.032	0.052
n	829		829		829	
Imputations	50		50		50	
F-test for model significance	108.08***		98.67***		76.33***	
Average RVI^b^	0.046		0.046		0.055	
Largest FMI^c^	0.073		0.062		0.091	
**Mean value of the O/E-ratio** ^d^						
England	0.996		1.008		1.011	
Finland	0.996		0.980		0.979	
Austria	1.003		1.006		1.004	

*** = *p* < 0.001; **= *p* < 0.01; *= *p* < 0.05

^a^ Robust standard errors

^b^ Average relative variance increase due to non-response

^c^ The largest fraction of missing information

^d^ The O/E-ratios were computed for those observations in data for which current SCRQoL was not missing (*n* = 805)

**Table 6:** Mean values of the risk-adjusted current SCRQoLs of carers in England, Austria and Finland using imputed data sets (*n* = 805)

Model B was used to compute risk-adjusted SCRQoL				
**Panel I**: Risk-adjusted current SCRQoL	Mean	Std. error		
England	0.736	0.013		
Finland	0.715	0.011		
Austria	0.734	0.011		
**Panel II** Pairwise testing for differences in risk-adjusted current SCRQoL means	Difference in means	Std. error	t-statistic^a^	*p*-value^b^
England vs. Finland	0.021	0.017	1.212	0.226
England vs. Austria	0.001	0.078	0.077	0.939
Finland vs. Austria	-0.019	0.016	-1.218	0.224

*** *p* < 0.001; ** *p* < 0.01; * *p* < 0.05

^a^ Appropriate t-statistic was chosen after testing equality of variances

^b^*p*-value of a two-sided t-test for equality of means

## Discussion

We have examined cross-country differences in informal carers’ risk-adjusted SCRQoL in Austria, England and Finland. SCRQoL was measured using the ASCOT-Carer four-level interview schedule [18, 19].

We found that the mean values of risk-adjusted SCRQoL of informal carers in England were 1.4–2.9% and 0.3–0.5% higher than the corresponding figures in Finland and Austria, and the mean values of risk-adjusted SCRQoL of informal carers in Austria were 0.8–2.7% higher than those values in Finland. The estimated cross-country differences in the mean values were small and non-significant. The small differences are somewhat at odds with our initial hypothesis, that was built on differences in the organisation, funding, and possibly also in the culture of LTC systems. The results are consistent with the observation that each of the studied countries provides different types of support to informal carers. The English support system had equal performance with Austrian or Finnish systems although informal carers in England more often co-resided with care recipients and were less healthy, which are risk factors of low SCRQoL.

Our descriptive findings indicate that informal carers in Austria, England and Finland differed in terms of risk-adjustment factors. The risk-adjustment models allowed us to control for such differences in carer and care recipient characteristics when drawing conclusions about the comparative performance of informal care support systems in three countries. However, reliable conclusions regarding the comparative performance of informal care support hinge on being able to choose appropriate risk-adjustment factors. To achieve this, we first excluded those factors from the estimated risk-adjustment models that affect the amount of support carers receive or their eligibility for services and benefits. Secondly, we applied statistical testing to choose the final risk-adjustment model for the comparative analysis. Without data on carers’ eligibility for informal care support or the take-up of benefits or unobserved but relevant risk-adjustment factors, some uncertainty remains whether the observed differences in risk-adjusted current SCRQoL can solely be explained by the country-specific support offered to informal carers.

We found that the current SCRQoL was positively related to the expected SCRQoL, SAH, and co-residence. These findings are in line with findings from previous literature analysing the SCRQoL of LTC service users and informal carers [44, 49, 50]. One might consider that our comparative results are driven by the strong positive association between the expected and current SCRQoL. To explore this possibility, we estimated the final risk-adjustment model without the expected SCRQoL. The estimated current SCRQoL ranking between the three countries remained the same, but the cross-country differences in the risk-adjusted current SCRQoL became larger. However, we decided to keep the expected SCRQoL in the risk-adjustment models as its inclusion is theoretically justified [49].

Our results come with some limitations. First, several carer and care recipient related variables suffered from missing values that reduced the number of observations used in the main analysis. In the sensitivity analysis, we applied multiple imputation of missing values to produce results that can be compared with those from the main analysis. Although some of the cross-country differences in risk-adjusted current SCRQoL became slightly larger using imputed data, the main findings remained unchanged suggesting that it is justified to rely on main findings using data with listwise deleted missing values. Second, even after imputation of missing values, relatively small country-specific samples may restrict generalisability of our findings to target populations of informal carers in Austria, England and Finland. Third, unobserved country-specific factors (such as cultural context of informal caregiving) and regulations may have influenced data collection, interviewees’ responses, and the final samples in practice. To minimise such effects as much as possible, we followed the study protocol developed in the English IIASC study to gather comparable information on non-institutional LTC service users and their informal carers in Austria and Finland. Finally, due to cross-section data, our comparative results reflect associations rather causal relationships between support systems and informal carers’ SCRQoL.

To conclude, the observed differences in the risk-adjusted current SCRQoL of informal carers in Austria, England and Finland were small, which is consistent with the observation that all three countries provide different types of support for informal carers. The support system in England performs equally with Austrian and Finnish systems although English informal carers are less healthy and co-reside with care recipients more often than carers in Austria or Finland in our sample, suggesting that the English system potentially performs better in supporting informal carers at such risk of low SCRQoL. Our results will enable national and local decisionmakers to benchmark their support systems for informal carers. Future studies should compare risk-adjusted SCRQoL in countries with distinctively different support systems for informal carers. Research is also needed to identify support characteristics that could enhance informal carers’ SCRQoL and enable the development of better support to improve carers’ well-being.

## Electronic supplementary material

Below is the link to the electronic supplementary material.

Supplementary Material 1
